# Surgical abortion service reorganization in response to the COVID-19 pandemic: a unique experience of attending second trimester D&E procedures under spinal anesthesia as emergency procedures

**DOI:** 10.3389/frph.2024.1426859

**Published:** 2024-09-18

**Authors:** Abraham Fessehaye Sium, Jaclyn M. Grentzer, Don Eliseo Lucero-Prisno III, Sarah Prager

**Affiliations:** ^1^ Department of Obstetrics and Gynecology, St. Paul’s Hospital Millennium Medical College-SPHMMC, Addis Ababa, Ethiopia; ^2^ Faculty of Management and Development Studies, University of the Philippines Open University, Los Baños, Philippines; ^3^ Department of Obstetrics and Gynecology, UW Medicine, Seattle, WA, United States

**Keywords:** COVID-19, service reorganization, abortion, second trimester abortion, D&E, surgical abortion

## Abstract

**Background:**

The COVID-19 pandemic created a massive shift in how health care systems interact with COVID testing for patients. To avoid delay in accessing second trimester surgical abortion at our hospital (St. Paul's Hospital Millennium Medical College) during this pandemic, dilation and evacuation (D&E) procedures were attended as emergency cases, instead of as elective surgical procedures, which then required adherence to the universal preoperative COVID-19 testing protocol. This study aimed at documenting the experience of this unique abortion service adjustment in response to the COVID-19 pandemic.

**Methods:**

This was a retrospective descriptive study conducted at St. Paul's Hospital Millennium Medical College (SPHMMC) in Addis Ababa, Ethiopia, from April 1, 2021 to July 31, 2021. We reviewed second trimester surgical abortion cases managed with D& E procedures, performed under spinal anesthesia using the emergency COVID-19 pre-operative testing protocol. Data were analyzed using SPSS version 23 and simple descriptive statistics were applied. Percentages and proportions were used to present the results.

**Results:**

Nineteen cases of second trimester D&E cases were reviewed. The median gestational age of the abortion clients was 21.57 weeks. Eight of 19 cases had cervical preparation with overnight intra-cervical Foley catheter placement while the rest 11 (57.9%) cases had their cervical preparation with Laminaria. The median time interval from initial evaluation of the abortion client to time of doing D& E procedure was 21.83 h.

**Conclusion:**

Surgical abortion service reorganization enabled abortion clients to access dilation and evacuation procedures within 24 h of their initial presentation. This prevented significant delays in accessing abortion care that could otherwise have occurred as a result of adhering to the preoperative COVID-19 testing protocols applied to elective surgeries.

## Introduction

Globally, 42 million abortions are performed each year and 10%–15% of the cases take place in the second trimester (1). The safety of both surgical and medical methods of abortion in the second trimester is well demonstrated worldwide (2). Surgical abortion involves preparing the cervix with mechanical or medical methods followed by dilation and evacuation (D&E) (3, 4). Dilatation and evacuation (D&E) is a brief surgical procedure to remove uterine contents for induced abortion or to remove tissues that remain in the uterus following miscarriage. Inhalational and intravenous anesthetics are widely used for this procedure (5).

The SARS-CoV-19 (COVID-19) viral pandemic created a massive shift in how health care systems interact with patients, staff, and the public, specifically with visitor policy changes, COVID testing for patients, and reduced or clustered care (6). A major concern during the COVID-19 lockdowns was reduced access to time-sensitive reproductive healthcare, in particular, healthcare related to abortions (7). To maintain access to abortion services during the pandemic, governments across Europe modified management of medical abortions by extending the gestational limit to 9 weeks in an ambulatory setting, authorizing telemedicine visits, and allowing direct pickup from pharmacies of call-in orders for mifepristone and misoprostol (8, 9). The focus for managing early pregnancy complications during the pandemic, was reported as geared toward safe outpatient conservative management wherever possible. In some miscarriages cases, manual vacuum aspiration under local or regional anesthesia was still preferred (10).

A review of preoperative COVID-19 testing protocols for elective surgery at 10 US institutions found that the protocols required universal preoperative COVID-19 testing 1–5 days before surgery, and all institutions postponed elective surgery in patients who tested positive for COVID-19 (11). St. Paul's Hospital and Millennium Medical College (Ethiopia) matched these protocols, requiring universal COVID-19 screening and obtaining a negative COVID-19 test result 1–5 days before proceeding with elective surgery and similarly experienced postponement of elective surgery in patients who tested positive for COVID-19. Initially, second trimester D&E procedures at an advanced gestational age (≥20 weeks) were designated to be performed in our Major OR as elective surgeries, where the described preoperative testing protocol applies. Later, a surgical abortion service reorganization was introduced with the purpose of managing these procedures without delay, due to sticking to the above-mentioned testing protocols. This intervention allowed D& E procedures to be done as emergency cases in the cesarean (CS) operating theater under spinal anesthesia, without the need to have a negative COVID-19 test but under strict COVID-19 infection prevention precautions. This study documents the benefits and safety of relying on this health service reorganization intervention amid the COVID-19 pandemic.

## Methods

### Study design, period, and setting

This study was a retrospective descriptive study done at St. Paul's Hospital Millennium Medical College (SPHMMC) in Addis Ababa, Ethiopia. SPHMMC is a center of excellence for family planning (where comprehensive abortion care and family planning service and training is provided) and it is also one of the leading tertiary teaching hospitals in Ethiopia. The study period was from April 1, 2021 to July 31, 2021, after implementation of the surgical abortion service reorganization defined as doing D&E procedures under spinal anesthesia on an emergency basis in the CS operation rooms, bypassing the requirement to have negative COVID-19 test results.

### Study population

All cases that had D&E under spinal anesthesia on an emergency basis in the CS OR theater, and for which information about reproductive characteristics and procedure-related variables was complete, were included in the study. Exclusion criteria included incomplete abortion, molar pregnancy, septic abortion, D&E performed with non-spinal anesthesia, and having incomplete information about reproductive characteristics and procedure related variables.

### Procedures

Abortion care clients undergoing D&E procedures were first evaluated at our outpatient clinic (MICHU clinic) by family planning fellows to assess eligibility for the procedure. After informed consent and laboratory test results were obtained and obstetric ultrasound finding were documented, overnight cervical preparation was carried out with mechanical methods (laminaria or intracervical Foley catheter placement) combined with mifepristone 200 mg orally. We defined intra-cervical foley catheter for cervical preparation as placement of 16–22 Fr sized Foley catheter balloon in to the cervical os with subsequent inflation of the balloon with 30–50 cc volume of normal saline, under aseptic technique using sterile vaginal speculum to identify the cervix, povidone iodine to clean the vaginal canal and cervix, and ring forceps to guide the tip of the Foley catheter into the cervical os.

Before administration of spinal anesthesia, clients were assessed for adequate cervical preparation to start the D&E procedure. They were pre-loaded with 1l of normal saline to minimize the risk of spinal anesthesia–induced hypotension. D&E procedures were started once the surgeon and anesthetist confirmed their impression of adequate pain control with the patients. Intraoperative ultrasonography was routinely utilized during the D&E procedures.

### Data collection and variables

Data were collected by reviewing maternal charts using a structured questionnaire prepared in English. We collected sociodemographic data, reproductive characteristics, abortion care characteristics, and outcomes of the cases. The completeness and consistency of the data was checked by the principal investigator. The primary outcome was time interval from initial evaluation of the abortion client to time of doing D& E procedure. The secondary outcomes were D&E procedure complications.

### Data analysis

Data were analyzed using using SPSS version 23. Simple descriptive statistics were used to analyze reproductive characteristics, and D& E procedure-related outcomes. Results were presented as percentages and frequencies. Due to poor documentation, procedure completion to client discharge time from the hospital was not included in the analysis as an outcome.

### Ethical considerations

Ethical clearance was obtained from the Institutional Board Review (IRB) of SPHMMC. The requirement for obtaining informed consent from study participants was waived by this ethics committee.

## Results

In this study, 19 cases of D&E met study inclusion criteria and were reviewed. Three subjects (15.8%) were teenagers (Table 1). Rape was the most commonly reported reason for seeking safe abortion care, represented in 42.1% of the cases followed by maternal health which accounted for 26.3% of the cases. The median gestational age and BPD measurement were 21.57 weeks and 5.15 cm respectively. More than half of the cases chose Nexplanon as a family planning method. The median time from initial client evaluation to D& E procedure was 21.83 h.

**Table 1:** Reproductive characteristics.

Variable	Category	*n*	%
Maternal age	≤18	3	15.8%
	>18	16	84.2%
Gravidity	Primigravida	13	68.4%
	Multigravida	6	31.6%
Parity	Nulliparous	11	61.1%
	Parous	7	38.9%
Indication for safe abortion	Rape	8	42.1%%
	Maternal health	5	26.3%
	Congenital anomaly	4	21.1%
	Incest	2	10.5%
Gestational age	Median	21.9	
BPD in cm	Median	5.15	
First evaluation to D and E procedure in hours	Median	21.83	
Family planning used	Nexplanon	10	52.6%
	Depo injection	1	5.3%
	IUD	3	15.8%
	COC	1	5.3%
	Declined	4	21.1%
Prior uterine scar	Yes	3	15.8%
	No	16	84.2%

Eight of 19 cases had cervical preparation with intra-cervical placement of Foley catheter (Table 2). There were no episodes of spinal anesthesia-induced hypotension or high spinal. There were no documented cases of hemorrhage, cervical tears, or uterine perforation. All procedures were attended by family planning fellows.

**Table 2:** Cervical preparation and D&E procedure characteristics.

Variable	Category	*n*	%
Cervical preparation methods	Intra-cervical Foley	8	42.1%
	Laminaria	11	57.9%
Spinal anesthesia-induced hypotension	BP ≤90/60 mm Hg	0	0%
High spinal		0	0%
PPH documented		0	0%
Cervical tear		0	0%
Uterine perforation		0	0%
D&E provider	Family planning fellow	19	100.0%

## Discussion

In the present study, the median time from first evaluation of the abortion clients to the time of doing D& E procedure was 21.83 h. According to the preoperative COVID-19 testing protocol for elective surgery at our study setting, accessing COVID-19 test results would normally have delayed those D&E cases at least 24 h before initiating cervical preparation, or even postponed their procedure by days to weeks if they tested positive for COVID-19. We found also that the majority of the clients had rape and maternal health as indications for abortion, which are arguably the most time-sensitive indications for safe abortion care.

It has been reported that access to abortion is desperately needed when pregnancy is the result of rape, both within and outside marriage (12). There is indirect evidence of potential harm from waiting periods once the woman decides to have an abortion, and many women find waiting periods burdensome (13). In line with these reports and various societal recommendations for the practice of abortion care during the COVID-19 pandemic, the authors argue that this vital health service reorganization (surgical abortion service reorganization) is an appropriate response to address the time-sensitive nature of abortion and sets a standard for how to practice surgical abortion care during the current and future pandemics.

In the 2020 American College of Surgeons COVID-19 Guidelines for Triage of Gynecology Patients, pregnancy termination (for medical indication or patient request) is classified under Surgeries that if significantly delayed could cause significant harm (14). In March 2020, the American College of Obstetricians and Gynecologists (ACOG), together with the American Association of Gynecologic Laparoscopists, the American Society for Reproductive Medicine, the Society of Family Planning, and the Society for Maternal-Fetal Medicine made the following joint statement; “While most abortion care is delivered in outpatient settings, in some cases care may be delivered in hospital-based settings or surgical facilities. To the extent that hospital systems or ambulatory surgical facilities are categorizing procedures that can be delayed during the COVID-19 pandemic, abortion should not be categorized as such a procedure. Abortion is an essential component of comprehensive health care. It is also a time-sensitive service.”

Our study found no major spinal anesthesia- or procedure-related complications. Currently, there is limited literature on use of spinal anesthesia for surgical abortion care. Ghomeishi, Ali et al. reported a positive experience of using spinal anesthesia for 25 elective abortion cases (15). A case report published in 2014 reported safe use of Modified spinal anesthesia with bupivacaine in patients with Eisenmenger syndrome undergoing surgical abortion (16). Aside from the small sample size, another limitation is unavailability of the time from D&E completion to hospital discharge. It would be helpful to know if spinal anesthesia delayed discharge as compared to other forms of procedural anesthesia for D&E.

Being among the first to report on second trimester D&E service reorganization during the COVID-19 pandemic and documenting the use of spinal anesthesia for D&E procedures are among the strengths of our study. Small sample size, retrospective data collection, and lack of further analysis of D&E outcomes according to cervical preparation methods (Laminaria vs. Foley catheter) are the main limitation of this study. Our findings are preliminary findings that require additional studies to have a firm clinical recommendation on wider use of spinal anesthesia for D&E procedures.

## Conclusion

Introduction of surgical abortion service reorganization (in response to the COVID-19 pandemic) enabled second trimester surgical abortion clients to access D&E services within 24 h of their initial presentation. It avoided a significant delay that could have otherwise resulted from adherence to the local preoperative COVID-19 testing protocols for elective surgery. This should remain a lesson for future pandemics and be noted as one of the opportunities created by the pandemic.

## Data Availability

The raw data supporting the conclusions of this article will be made available by the authors, without undue reservation.
